# Small RNA-Seq Reveals Similar miRNA Transcriptome in Children and Young Adults with T-ALL and Indicates miR-143-3p as Novel Candidate Tumor Suppressor in This Leukemia

**DOI:** 10.3390/ijms231710117

**Published:** 2022-09-04

**Authors:** Małgorzata Dawidowska, Natalia Maćkowska-Maślak, Monika Drobna-Śledzińska, Maria Kosmalska, Roman Jaksik, Donata Szymczak, Małgorzata Jarmuż-Szymczak, Alicja Sadowska-Klasa, Marzena Wojtaszewska, Łukasz Sędek, Tomasz Wróbel, Jan Maciej Zaucha, Tomasz Szczepański, Krzysztof Lewandowski, Sebastian Giebel, Michał Witt

**Affiliations:** 1 Institute of Human Genetics, Polish Academy of Sciences, 60-479 Poznan, Poland; 2 Department of Systems Biology and Engineering, Silesian University of Technology, 44-100 Gliwice, Poland; 3 Department and Clinic of Hematology, Blood Neoplasms, and Bone Marrow Transplantation, Wroclaw Medical University, 50-367 Wroclaw, Poland; 4 Department of Hematology and Bone Marrow Transplantation, Poznan University of Medical Sciences, 60-569 Poznan, Poland; 5 Department of Hematology and Transplantology, Medical University of Gdańsk, 80-210 Gdansk, Poland; 6 Department of Microbiology and Immunology, Zabrze, Medical University of Silesia in Katowice, 40-055 Katowice, Poland; 7 Department of Pediatric Hematology and Oncology, Zabrze, Medical University of Silesia in Katowice, 40-055 Katowice, Poland; 8 Department of Bone Marrow Transplantation and Onco-Hematology, Maria Sklodowska-Curie National Research Institute of Oncology, Gliwice Branch, 44-102 Gliwice, Poland

**Keywords:** T-cell acute lymphoblastic leukemia (T-ALL), adolescents and young adults (AYA), miR-143-3p, tumor suppressor miRNA, targeting FGFR signaling, miRNA-seq, target prediction, pathway analysis, personalized medicine

## Abstract

We aimed to identify miRNAs and pathways specifically deregulated in adolescent and young adult (AYA) T-ALL patients. Small RNA-seq showed no major differences between AYA and pediatric T-ALL, but it revealed downregulation of miR-143-3p in T-ALL patients. Prediction algorithms identified several known and putative oncogenes targeted by this miRNA, including *KRAS*, *FGF1*, and *FGF9*. Pathway analysis indicated signaling pathways related to cell growth and proliferation, including FGFR signaling and PI3K-AKT signaling, with the majority of genes overrepresented in these pathways being predicted targets of hsa-miR-143-3p. By luciferase reporter assays, we validated direct interactions of this miRNA with *KRAS*, *FGF1* and *FGF9*. In cell proliferation assays, we showed reduction of cell growth upon miR-143-3p overexpression in two T-ALL cell lines. Our study is the first description of the miRNA transcriptome in AYA T-ALL patients and the first report on tumor suppressor potential of miR-143-3p in T-ALL. Downregulation of this miRNA in T-ALL patients might contribute to enhanced growth and viability of leukemic cells. We also discuss the potential role of miR-143-3p in FGFR signaling. Although this requires more extensive validation, it might be an interesting direction, since FGFR inhibition proved promising in preclinical studies in various cancers.

## 1. Introduction

T-cell acute lymphoblastic leukemia is an aggressive hematological malignancy arising from T-cell precursors, diagnosed in all age groups. The group of adolescents and young adults (AYA) is most frequently defined by 15–39 years of age at diagnosis [1]. The transitional age of AYA patients means that younger patients (<18 years of age) are treated in pediatric centers according to pediatric-oriented protocols, while those over the age of 18 are treated in the centers for adults with either pediatric-inspired or adult-oriented regimens. Recent studies have demonstrated that ‘pediatric’ protocols are more effective for AYA patients [1,2,3]. However, even with pediatric-inspired regimens, treatment outcomes in the AYA group are worse than in children (overall survival rates of approx. 60% vs. approx. 90% in children) [2]. This implies a need for better understanding of the biology of T-ALL in the AYA group, to develop more efficient treatment strategies. The identification of biological processes specifically deregulated in AYA T-ALL might pave the way toward targeted therapies, e.g., inhibitors of activated signaling pathways [4].

The current knowledge on the molecular characteristics of AYA T-ALL is limited. There are a few studies using next-generation sequencing to characterize the genomic landscape of T-ALL in children and adults [5,6,7], none of them specifically focusing on young adults. Whole exome sequencing revealed 2.5 times higher mutation burden (somatic protein-altering SNVs and indels) in adult patients (>15 years) than in children and different frequencies of mutations in driver genes between adults and children [5]. RNA-seq revealed a similar number of gene fusions in children (<18 years of age) and adults (>18 years), yet with lower diversity of fusion types among adults than children [6]. These results indicate differences in the biology of T-ALL among age groups.

miRNA expression profiles and the global miRNA transcriptome have already been investigated in T-ALL by several groups, as reviewed by Correia et al. [8], including in our group [9]. However, it has not been investigated thus far in AYA T-ALL. Since miRNAs are implicated in post-transcriptional gene regulation, aberrantly expressed miRNAs may act as oncogenes (overexpressed miRNAs repressing tumor suppressor genes) or as tumor suppressors (underexpressed miRNAs insufficiently repressing oncogenes). miRNAs affecting essential signaling pathways, cell proliferation, and apoptosis contribute to cancer development and progression. Comprehensive investigation of miRNA transcriptome and miRNAs’ involvement in cellular functions might indicate novel mechanisms of leukemia biology, potentially specific to different patient subsets.

We investigated global miRNA expression by small RNA-seq, followed by in silico prediction of miRNA target genes and their involvement in biological pathways and processes. We aimed to identify miRNAs specifically expressed in AYA T-ALL and to focus on those with potential therapeutic implications. Small RNA-seq demonstrated no considerable differences between AYA and pediatric T-ALL. An important finding is the downregulation of hsa-miR-143-3p in pediatric T-ALL patients. We demonstrated its tumor suppressor potential as shown by the restriction of cell proliferation upon overexpression of this miRNA in T-ALL cells in vitro. We also validated direct interaction of hsa-miR-143-3p with *KRAS*, *FGF1* and *FGF9*, involved in FGFR (fibroblast growth factor receptor) pathway. Although the roles of this miRNA and its target genes in T-ALL biology need more extensive validation, we discussed the implications of our findings in light of the potential application of FGFR inhibition in this leukemia.

## 2. Results

We investigated miRNA transcriptome in the AYA T-ALL group as compared to pediatric T-ALL and controls (previously published [9]). In total, we analyzed 71 T-ALL cases: 19 AYA (15–39 years of age) and 52 children (<15 years of age), five control samples (normal T-cells of healthy bone marrow donors <18 years of age) and six samples of normal thymocytes (CD34^+^ and CD4^+^CD8^+^). miRNA-seq results of pediatric and AYA patients were analyzed according to the same methodology. Three samples of pediatric T-ALL from our previous study focusing on pediatric T-ALL, were resequenced as ‘inter-experimental calibrators’ in the current study. Additionally, we applied the correction for batch effect, to enable a joint analysis of all miRNA-seq results. Characteristics of samples are presented in Table S1.

### 2.1. Overview of miRNA Transcriptome in AYA Patients

Small RNA-seq in 19 AYA T-ALL samples resulted in an average number of 10.5 million reads mapped to mature miRNAs/sample (range: 1.7–26.4 million reads) (Table S2). In total, we identified 1244 miRNAs expressed in AYA T-ALL samples (with at least two reads/sample); 1148 known miRNAs and 96 candidate novel miRNAs (not previously reported in miRBase22) (Table S3). The 10 most highly expressed miRNAs in AYA patients included: hsa-let-7a-5p/7c-5p, hsa-let-7f-5p, hsa-miR-92a-3p, hsa-miR-26a-5p, hsa-miR-21-5p, hsa-miR-128-3p, hsa-let-7i-5p, hsa-miR-181a-5p, hsa-let-7g-5p, hsa-miR-16-5p (Figure S1). This list largely resembles that of which we previously described in pediatric T-ALL patients [9].

### 2.2. miRNA Transcriptome Does Not Clearly Discriminate AYA and Pediatric T-ALL

**Figure 1:**
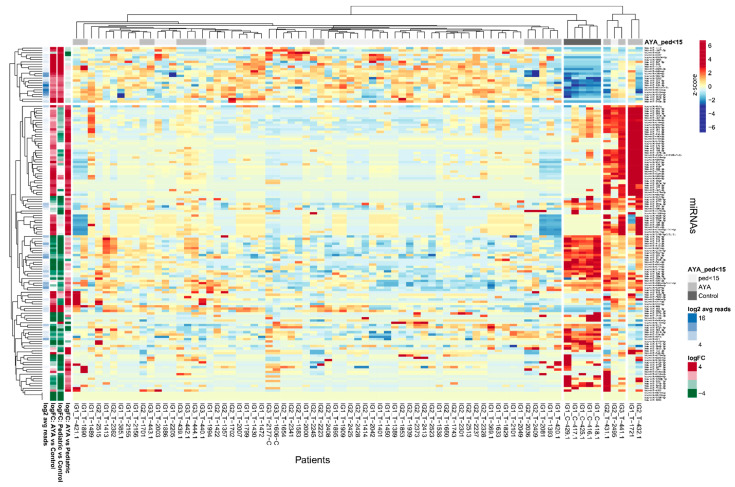
Differentially expressed miRNAs in AYA T-ALL, pediatric T-ALL and control samples. Heatmap and dendrograms of Z-score normalized miRNA expression levels created for miRNAs differentiating T-ALL samples of AYA, pediatric and normal controls (bone marrow T-cells). Rows represent miRNAs; columns represent samples. Dendrograms are based on complete-linkage hierarchical clusterization and Euclidean distances. The fold changes and the abundance of miRNAs (in log scale) are shown on the left side of the plot.

To identify miRNAs specifically expressed in AYA patients, which could indicate potentially targetable AYA-specific features of leukemia, we investigated miRNAs differentially expressed between AYA vs. pediatric group as well as miRNAs differentially expressed between T-ALL and controls (Figure 1). Hierarchical clustering method showed that discrimination between AYA and pediatric T-ALL, based on the miRNA transcriptome, is weak: samples of AYA and pediatric patients do not group separately under the major branches of the dendrogram. miRNA expression profile differs mostly between T-ALL (both AYA and pediatric samples) and controls (Figure 1). The PCA plot (Figure S2) also shows that miRNA expression profile does not clearly discriminate between AYA and pediatric patients and is largely similar in both age groups as compared to controls in our study.

Out of 1244 miRNAs expressed in AYA T-ALL samples, only 88 miRNAs were differentially expressed between AYA and pediatric patients, including 11 candidate novel miRNAs (Table S3). Among differentially expressed miRNAs, three were downregulated in AYA vs. pediatric patients (hsa-miR-4713-5p, hsa-miR-639, and hsa-miR-6724-5p, the latter was also differentially expressed between AYA and controls), and 85 miRNAs were upregulated in AYA vs. pediatric samples. The upregulated miRNAs included six miRNAs, which were not only differentially expressed between AYA vs. pediatric samples but additionally between pediatric vs. control samples (hsa-miR-143-3p, hsa-miR-151a-3p, hsa-miR-4420-3p, hsa-miR-4728-3p, hsa-miR-4749-3p, novel_miRNA_chr17:7306866-7306887).

We further focused on miRNAs that differentiated AYA T-ALL from pediatric T-ALL and simultaneously differentiated T-ALL from the controls. We identified only seven such miRNAs (Table 1), all but one (hsa-miR-6724-5p) showing upregulation in AYA vs. pediatric patients. Of note, only this particular miRNA was simultaneously differing AYA vs. control samples; the remaining six miRNAs were discriminative between AYA vs. pediatric and pediatric vs. controls (Table S3). The low number of miRNAs specifically expressed in the AYA group is in line with the observations from hierarchical clustering, indicating that the miRNA transcriptome is similar in both age groups.

### 2.3. Target Prediction and Pathway Analysis Indicate Deregulation of Cancer-Related Signaling Pathways

To investigate the potential involvement of miRNAs differentially expressed between AYA and pediatric T-ALL samples and controls, we performed in silico target prediction for all miRNAs listed in Table 1, with the exclusion of a novel miRNA identified in our miRNA-seq. We used eight miRNA target prediction algorithms (DIANA-microT, ElMMo, MicroCosm, miRanda, miRDB, PicTar, PITA, TargetScan). We also searched three repositories of experimentally validated miRNA:mRNA interactions (miRecords, miRTarBase, DIANA-TarBase). In addition, we searched miR2Disease, PhenomiR, and PharmacomiR, providing data on the potential involvement of miRNAs in diseases and drug response (Table S4).

**Figure 2:**
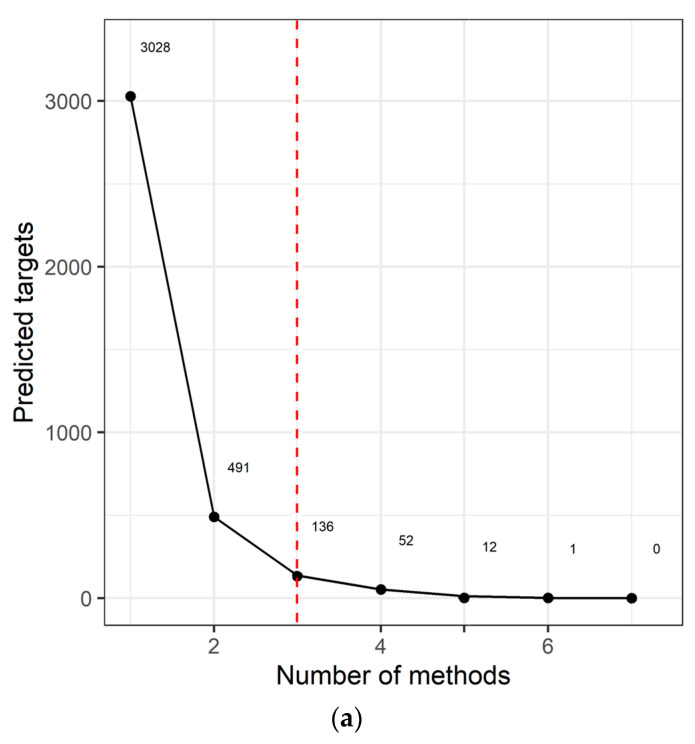

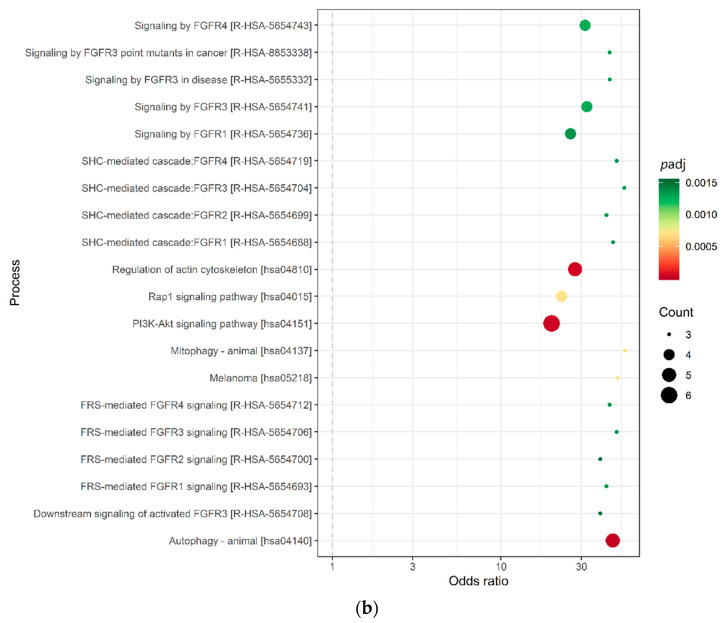
miRNA target prediction and pathway analysis. (**a**) Number of consistent target gene predictions for miRNAs specifically expressed in AYA T-ALL in relation to the number of prediction algorithms used. (**b**) Processes and pathways potentially affected by miRNAs specifically expressed in AYA T-ALL, revealed by overrepresentation analysis. The plot presents odds ratios for the selected terms, identified using conditional hypergeometric test, with Benjamini and Hochberg correction for multiple testing and 0.05 significance level. The size of the dots (Count) represents the number of genes (predicted targets of the studied miRNAs) involved in a given biological process; the color of the dots represents *p* value (*p* adj) adjusted for multiple testing.

We identified 3028 genes potentially repressed by miRNAs specifically deregulated in the AYA T-ALL group (Table S4). For 136 target genes, consistently identified by at least three prediction algorithms (Figure 2a), we analyzed their overrepresentation in the processes and pathways, defined by the Gene Ontology, KEGG, and Reactome databases (Figure 2b; Table S4). Genes targeted by miRNAs deregulated in AYA T-ALL were significantly overrepresented in several pathways known to be related to leukemia and cancer in general. These include: Rap1 (Ras-associated protein-1) signaling, PI3K-Akt (phosphatidylinositol 3-kinase) pathway, and FGFR signaling (FGFR1-4), the latter clearly stands out from this analysis. The vast majority of the genes overrepresented in these processes are predicted to be targets of miR-143-3p (Table S4), highlighting the potential importance of this miRNA in the biology of T-ALL cells.

### 2.4. miR-143-3p Affects Proliferation of T-ALL Cells In Vitro

In miRNA-seq, miR-143-3p showed downregulation in T-ALL patients, which we confirmed by RT-qPCR (Appendix A). Prediction algorithms and overrepresentation analysis revealed several oncogenes potentially targeted by this miRNA, including *KRAS*, *FGF1*, *FGF9*, *ITGA6*, and *MAP3K7* (Table S4). Insufficient repression of oncogenes by miR-143-3p might confer growth-promoting effects in T-ALL cells.

**Figure 3:**
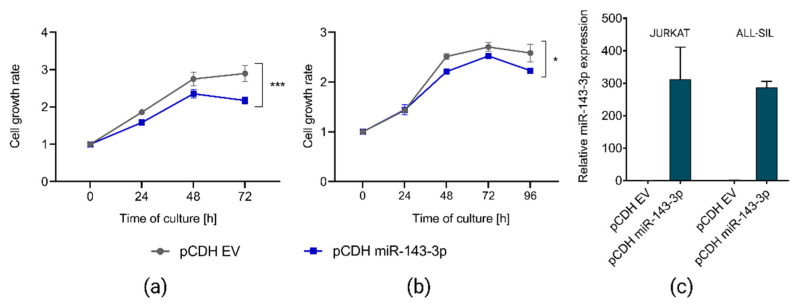
Effects of overexpression of miR-143-3p in JURKAT and ALL-SIL T-ALL cell lines. Decreased growth of JURKAT (**a**) and ALL-SIL (**b**) cells, as demonstrated by CCK8 assay. Cell growth rates were calculated as fold change of OD450 for each examined time point in reference to the starting point (0 h). pCDH EV, empty vector used as a control; *** *p* < 0.001; * *p* < 0.05 (*p*-value calculated by two-way ANOVA for miR-143-3p expression as independent variable). RT-qPCR results demonstrating overexpression of miR-143-3p in both T-ALL cell lines upon transduction with pCDH miR-143-3p overexpression vector (**c**).

To address our hypothesis on the tumor suppressor role of miR-143-3p and oncogenic effects of its downregulation, we selected two T-ALL cell lines (JURKAT and ALL-SIL) showing low endogenous expression of this miRNA and transduced them to overexpress miR-143-3p. In both cell lines, we observed statistically significant reduction of cell growth upon miR-143-3p overexpression as compared to control (Figure 3), indicating the tumor suppressor potential of this miRNA.

### 2.5. miR-143-3p Interacts with FGF1, FGF9 and KRAS

**Figure 4:**
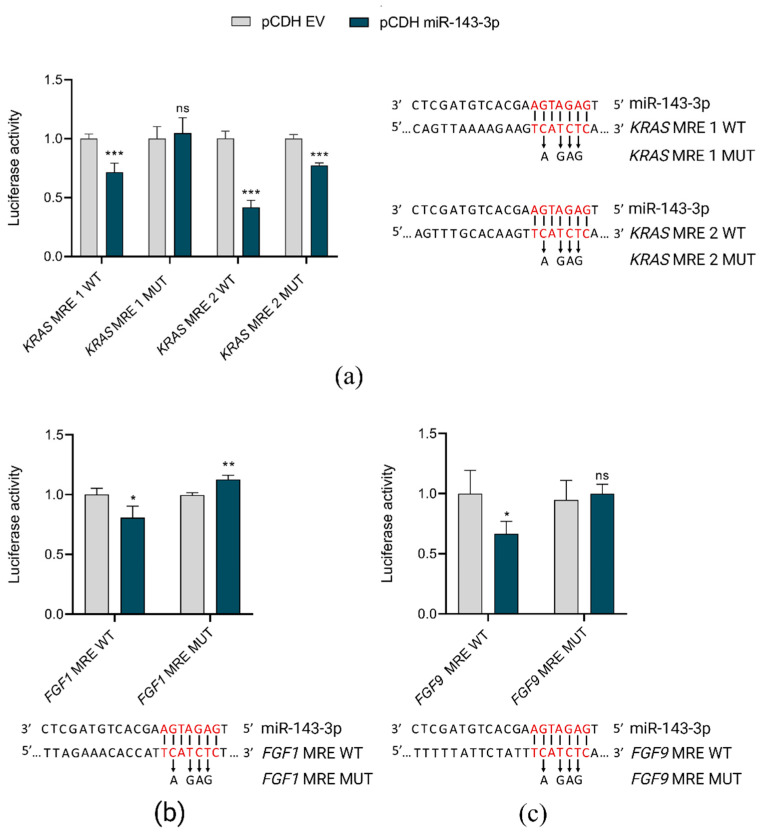
In vitro validation of direct interactions of miR-143-3p with *FGF1*, *FGF9* and *KRAS*. pCDH EV, pCDH empty vector; pCDH miR-143-3p, pCDH vector containing miR-143-3p coding sequence MRE, miRNA responsive element in 3′UTR of target gene; WT, wild-type sequence; MUT, sequence with mutations introduced within MRE; *** *p* < 0.001; ** *p* < 0.01; * *p* < 0.05; ns, not significant. The graphs present the decrease in relative luciferase activity in the presence of miR-143-3p in reference to control (empty vector). For each graph, the predicted interaction sites of miR-143-3p with MREs are shown, with indication of nucleotides mutated in the rescue experiment. (**a**) Interactions between miR-143-3p and two MREs in *KRAS* gene. (**b**) Interaction between miR-143-3p and MREs in *FGF1* gene. (**c**) Interaction between miR-143-3p and MREs in *FGF9* gene.

To validate in silico predictions of miR-143-3p target genes, we used luciferase reporter assays. Co-transfection of HEK293T cells with pCDH vector coding miR-143-3p and pmiRGLO plasmids, containing miRNA responsive elements (MREs) in the 3′UTRs of selected target genes (*FGF1*, *FGF9* and *KRAS*) resulted in the reduction of reporter luciferase activity as compared to controls (co-transfection with pCDH empty vector). These direct interactions of miR-143-3p and its target genes were lost when mutations were introduced into MREs (Figure 4).

## 3. Discussion

The major finding of the study is the similarity of miRNA transcriptome in pediatric and AYA patients. An additional but potentially important observation is the downregulation of miR-143-3p in T-ALL patients and its putative involvement in leukemia biology as shown by our in silico predictions and basic functional experiments. Among predicted targets of this miRNA, there are several oncogenes significantly overrepresented in signaling pathways of potential importance for T-ALL cells (Figure 2b). miR-143-3p and its target genes (*FGF1*, *FGF9*, *KRAS*) clearly stand out from this overrepresentation analysis (Table S4). Since these genes are implicated in cell proliferation, apoptosis, and stress responses, we hypothesized that insufficient negative regulation over these oncogenes by downregulated miR-143-3p might serve as an oncogenic mechanism in this leukemia. We demonstrated that overexpression of this miRNA in two T-ALL cell lines resulted in the reduction of cell growth. We also validated direct interaction of miR-143-3p with *FGF1*, *FGF9,* and *KRAS.*

The tumor suppressor role of miR-143-3p has not been reported thus far in the context of T-ALL. However, it has been suggested in several cancer types, including solid tumors [10], myeloid and B-cell malignancies [11,12,13]. In acute lymphoblastic leukemia originating from B-cell precursors (BCP-ALL), miR-143 was shown to be downregulated in diagnostic and relapse samples as compared to remission samples [11]. The authors tested the hypothesis on the tumor suppressor role of this miRNA by cell growth assays in REH cells upon miRNA overexpression, yet no impact on growth rate was observed in this particular B-ALL cell line. The downregulation of miR-143 was concluded as a potential biomarker of leukemic cells, but the underlying mechanisms remained unresolved. Similar results were reported in another study in ALL patients, showing downregulation of miR-143 in diagnostic samples and then increasing miRNA levels along with decreasing burden of leukemic cells upon induction treatment [14]. This study focused purely on clinical utility of miRNA expression, and no functional studies were included to investigate the underlying mechanisms. miR143-3p has been shown to be downregulated in colon cancer samples; upregulation of miR143-3p by transfection of HT-29 colon cancer cell line resulted in decreased expression of ERK5 and its upstream activator, KRAS protein [15]. Downregulation of miR143-3p has also been observed in breast cancer samples; upregulation of this miRNA in the MCF-7 cell line resulted in decreased expression of ERK5 and MAP3K7 proteins, and decreased cell viability [16].

Our pathway analysis revealed several genes recurrently overrepresented in signaling pathways of potential importance for cell survival, proliferation, and drug resistance, including *FGF1*, *FGF9*, *KRAS,* and *MAP3K7,* all implicated in FGFR signaling (Figure 2b; Table S4). Three major downstream pathways of FGFR signaling include PI3K-AKT, STAT and Ras-MAPK signaling [17], all three reported to be deregulated in T-ALL. The latter pathway involves KRAS, MAP3K7, and ERK5 (alias MAPK7), all shown to be regulated by miR-143 in various solid tumors, yet not in T-ALL, thus far.

FGFR signaling is crucial in embryonic development and in adult organism, by the involvement in cell proliferation, cell growth, morphogenesis, angiogenesis, tissue repair and metabolism [18]. Dysregulation of the FGFR pathway due to *FGFR* gene amplification, overexpression, mutations and rearrangements with at least 15 fusion partner genes has been reported in many cancers, including hematological malignancies [19,20]. In BCP-ALL patients, mutations in *FGFR1*, *FGFR2*, *FGFR3* are rare (<1%), as shown by targeted next-generation sequencing [19]. The frequency of *FGFR* mutations in T-ALL is also low; *FGFR3* and *FGFR4* mutated in approx. 5% of patients, as reported by Rokita et al. [21]. In our unpublished whole genome sequencing dataset, mutations of *FGFR* genes are also infrequent; *FGFR2* mutated in 1.5% and *FGFR3* in 3% of pediatric T-ALL patients. These data indicate that there are other mechanisms behind upregulation of this pathway. Thus far, there have been no reports on the overactivation of this pathway in T-ALL by aberrantly expressed miRNAs. However, miRNAs have been shown to affect this pathway in other cancer types [22,23].

FGFR pathway is considered as a therapeutic target—several small molecule inhibitors targeting FGFR receptor tyrosine kinases have been already approved or in clinical trials, both in solid tumors and in hematological malignancies (*ClinicalTrials.gov identifier: NCT03011372*) [24,25]. These include ponatinib (AP24534) a pan-FGFR inhibitor, targeting all members of the FGFR family (FGFR-1, -2, -3, -4). It has been tested in solid tumors and hematological malignancies, including T-ALL, used in combination with PIM inhibitors [20,26,27,28,29]. There is also an ongoing trial on the use of another FGFR inhibitor, pemigatinib (INCB054828) in patients with myeloid/lymphoid neoplasms with FGFR1 rearrangement (*ClinicalTrials.gov identifier: NCT03011372*).

However, oncogenic overactivation of FGFR signaling pathway might not only be due to aberrations affecting the receptor tyrosine kinase (FGFR), but also due to overexpression of the ligands (FGFs, fibroblast growth factors, such as *FGF1* or *FGF9*) or other components of this pathway, downstream of FGFR (such as *KRAS*).

FGF family proteins exert broad mitogenic and pro-survival activities; in cancers, they have been implicated in tumor growth, invasion and resistance to anticancer therapies [30]. FGFs are abundantly expressed by bone marrow stromal cells and secreted in exosomes, which are then endocytosed by leukemia cells, contributing to resistance to tyrosine kinase inhibitors (TKIs) [31]. Although T-ALL originates in the thymus, FGFR signaling might create a leukemia-protective microenvironment for T-ALL cells infiltrating the bone marrow niche, similarly to the effects of FGFR signaling demonstrated for AML cells [31,32].

KRAS is a member of GTPase superfamily of proteins, acting as an ‘on/off switch’ molecule in the transduction of signals from growth factors; thus, it is involved in several signaling pathways [33]. Oncogenic activation of *KRAS* is implicated in many different cancers, mainly by mutations in *KRAS* [34]. However, mutations affecting this proto-oncogene are not the only mechanism of its activation. Recently, *KRAS* has been shown to be regulated by non-coding RNAs (lncRNAs, miRNAs and circRNAs) in the context of cancer development [35].

Insufficient repression of FGFR pathway components by miR-143-3p might potentially contribute to survival advantage of leukemic cells. Downregulation of this miRNA might serve as a mechanism of oncogenic activation of this pathway, alternative to aberrations affecting FGFR receptor tyrosine kinases, typically screened to select patients who could benefit from FGFR-inhibiting therapies.

### Limitations

Although we showed miR-143-3p as a novel candidate tumor suppressor affecting T-ALL cell proliferation in vitro, the underlying mechanisms need extended validation. It would be important to distinguish if the reduction of proliferation rate of T-ALL cells, observed upon forced overexpression of this miRNA, stems from induction of apoptosis or impairment of proliferation. By dual luciferase reporter assays, we validated in silico predictions of *FGF1*, *FGF9,* and *KRAS* as targets for miR-143-3p. However, these assays demonstrate in vitro interactions of miRNAs with MREs in 3′UTRs of the target genes in an artificial experimental setting. The functional impact of miR-143-3p on deregulation of FGFR signaling in T-ALL cell lines and patients should ideally be verified by a proteomic approach, enabling a global overview of proteins and pathways affected by this miRNA.

## 4. Materials and Methods

### 4.1. T-ALL Primary Samples and Controls

T-ALL samples of all patients were obtained at initial diagnosis. Samples of patients aged < 18 years were collected at the centers of Polish Pediatric Leukemia and Lymphoma Study Group. Details of sample preparation, including RNA isolation and quality control were described previously [9]. Samples of patients aged ≥ 18 years were collected in centers of Polish Adult Leukemia Group. Control samples of 5 healthy unrelated bone marrow donors, aged < 18 years were subjected to isolation of mononuclear cells using density gradient centrifugation followed by immunomagnetic separation using Human T Lymphocyte Enrichment Set-DM (Becton Dickinson, Franklin Lakes, NJ, USA) to obtain normal T-cells as controls. Thymocyte CD4+ CD8+ and CD34+ samples, obtained from 3 children undergoing cardiac surgery, were also used as controls. CD34+ thymocyte samples were obtained using MACS purification with CD34 microbeads (Miltenyi Biotec, Bergisch Gladbach, Germany); CD4+ CD8+ thymocytes were obtained by CD4 and CD8 labeling and sorting by a FACSAriaIII (BD Biosciences, Franklin Lakes, NJ, USA), as previously published [36,37]. Characteristics of samples are presented in Table S1. This includes immunophenotypic EGIL classification of T-ALL samples and classification into genetic subtypes based on an oncogenic activation of the following genes *TLX1*, *TLX3*, *TAL1*, *TAL2*, *LMO1*, *LMO2*, *LYL1*, and *HOXA* genes. Genetic subtypes were defined based on gene expression levels and fusions identified using RNA-seq (Array Express E-MTAB-11759). RNA-seq results were available for the majority of patients < 18 years at diagnosis and for two patients > 18 years of age (Appendix B).

### 4.2. T-ALL Cell Culture In Vitro

HEK293T cells were cultured under standard conditions in Dulbecco’s modified Eagle’s medium (Gibco, Thermo Fisher Scientific, Waltham, MA, USA) with 10% fetal bovine serum (Gibco, Thermo Fisher Scientific) and 1% penicillin/streptomycin solution (Sigma Aldrich, St. Louis, MO, USA). T-ALL cells were cultured under standard conditions in RPMI-1640 medium (Gibco, Thermo Fisher Scientific) with 10% or 20% of fetal bovine serum (Gibco, Thermo Fisher Scientific) for JURKAT and ALL-SIL, respectively.

### 4.3. Small RNA Sequencing and Bioinformatics Analyses

In this study, we used small RNA-seq results of pediatric T-ALL (aged < 18) and control samples, which we obtained previously; data are available in the ArrayExpress database (http://www.ebi.ac.uk/arrayexpress) under accession number E-MTAB-7446 [9]. Patients from this first batch, aged < 15 years, were analyzed as pediatric T-ALL in this study, while patients aged 15–18 years were analyzed in the AYA group. The remaining samples were sequenced in two following batches. The proportion of pediatric and AYA patients in each batch is as follows: batch I (28 pediatric, 6 AYA), batch II (27 pediatric, 7 AYA), batch III (3 pediatric, 6 AYA). The same 3 pediatric samples were used as ‘interexperimental calibrators’ in sequencing of all three batches, to enable normalization and correction for batch effect using edgeR. In the final analyses, these 3 samples (sequenced in 3 batches) were represented by only one replicate each (samples with the highest normalized read counts were used). The effectiveness of batch effect correction is presented in PCA plots demonstrating clustering of samples from all 3 batches before and after correction (Figure S2).

In brief, in batch I, the libraries were generated with NEBNext Multiplex Small RNA Library Prep Set for Illumina (New England Biolabs, Ipswich, MA, USA), quality controlled based on size distribution and concentration using 2100 Bioanalyzer (Agilent Technologies, Santa Clara, CA, USA), and sequenced using NextSeq500 Illumina and standard settings: 10 million reads/per sample, read length: 51 bp single-end. FASTQ files for each sample were generated using the bcl2fastq software v2.20 (Illumina, San Diego, CA, USA). NGS service was performed by Exiqon (Exiqon, Vedbæk, Denmark). Sequencing of samples in batches II and III was conducted at QIAGEN Genomic Services (Düsseldorf, Germany). Libraries were prepared using QIAseq miRNA Library Kit, including the ligation of adapters containing unique molecular identifiers, UMIs (QIAGEN). Quality control of libraries preparation was performed using 2100 Bioanalyzer (Agilent Technologies, Santa Clara, CA, USA). Based on the quality of the inserts and the concentration measurements, the libraries were pooled in equimolar ratios. The library pools were quantified using qPCR. The library pools were then sequenced on a NextSeq 500 Illumina using standard settings. Raw data were de-multiplexed, and FASTQ files were generated using the bcl2fastq software (Illumina, San Diego, CA, USA).

Quality control of reads was conducted using FastQC ver. 0.11.5 (http://www.bioinformatics.babraham.ac.uk/projects/fastqc, accessed on 8 January 2019), FastQ Screen ver. 0.5.1 [38], and custom data visualization scripts. Raw sequencing reads were adapter-trimmed using Cutadapt [39] (ver. 1.11) and aligned with Bowtie [40] (ver. 1.2.2) to a modified version of miRBase (ver. 22) created according to the miRge specifications [41]. We used an iterative alignment of reads: reads were first aligned to mature miRNA sequences (miRBase ver. 22); unaligned reads were sequentially matched against hairpin miRNAs (miRBase ver. 22), noncoding RNAs, (Ensembl cDNA database), and again to mature miRNA sequences (miRBase ver. 22) using less stringent criteria [41]. Detection of candidate novel miRNAs was based on miRge2 for each individual sample. The results were later combined between samples based on genomic coordinates of identified miRNAs (partial overlap was considered sufficient). Both known and candidate novel miRNAs, of which at least two reads were aligned in a single sample, were further analyzed. Read normalization and identification of differentially expressed miRNAs, was conducted using edgeR [42], accounting for batch effect by specifying an appropriate model matrix with Benjamini and Hochberg correction for multiple testing and 0.05 significance level. Read counts used in the tests were normalized in edgeR using Trimmed Mean of Mvalues (TMM) algorithm. If more than two groups existed in a specific classification method, we conducted pairwise comparisons between them for each unique combination and additionally performed an ANOVA test independently for each miRNA with Benjamini and Hochberg correction for multiple testing. ANOVA was conducted using edgeR normalized data after batch effect correction based on ComBat [43]. Results of all analyses were presented as heatmaps of Z-score normalized miRNA expression levels with dendrograms based on complete-linkage hierarchical clusterization and Euclidean distances.

### 4.4. RT-qPCR Validation of miRNAs’ Expression

RT-qPCR validation of expression of hsa-miR-143-3p, hsa-miR-151-3p, hsa-miR-582-5p and hsa-miR-6724-5p was performed as described previously [9]. Briefly, RNA samples were reverse transcribed with TaqMan Advanced miRNA cDNA Synthesis Kit (Thermo Fisher Scientific). TaqMan Fast Advanced Master Mix, predesigned TaqMan Advanced miRNA assays (Thermo Fisher Scientific), and 7900HT Fast Real-Time PCR System (Applied Biosystems, Waltham, MA, USA) were used. Relative quantification of expression was performed using comparative delta CT method (ΔΔ CT) [44] based on three endogenous normalizer miRNAs (hsa-miR-16-5p, hsa-miR-25-3p and hsa-let-7a-5p) as described previously [45]. Two-tailed Student’s t test was used to test for the significance of differences in expression between T-ALL samples and controls, with *p* < 0.05 for statistical significance.

### 4.5. miR-143-3p Overexpression in T-ALL Cells In Vitro

For assembly of lentiviral particles, HEK293T cells were seeded on a 6-well culture plate. Upon 70–80% confluence, the cells were transfected with 600 ng of each: pRSV.REV, pMSCV-VSV-G and pMDLg/PRRE packing vectors and 1200 ng of transfer vector. Transfection was performed using JetPrime DNA/siRNA Transfection Kit (Polyplus Transfection, New York, NY, USA). After 24 h, the transfection medium was replaced with 1 mL fresh medium. After 48 h, the medium was collected and filtered with 0.45 µm filters. For transduction, cells were seeded on a 6-well plate in 1.8 mL of RPMI-1640 medium. Then, 200 µL of filtered medium containing lentiviral particles was added to each well. Polibrene Reagent (Sigma Aldrich, St. Louis, MO, USA) was added to each well at the final concentration of 8 µg/mL. Cells were spinfected for 90 min (at 1500 rpm and 32 °C). Antibiotic selection of transduced cells was started 3–4 days post transduction. For selection, puromycin dihydrochloride (Gibco, Thermo Fisher Scientific) was used at the concentration of 10 µg/mL. The selection procedure was conducted for 7 days. The effectiveness of transduction was assessed with the use of CytoFlex S flow cytometer (Beckman Coulter, Indianapolis, IN, USA) with GFP as a marker.

For miR-143-3p overexpression, the pre-miRNA sequence was amplified and cloned into pCDH-CMV-MCS-EF1-GreenPuro overexpression vector (System Biosciences, Frontstraat, The Netherlands). For Dual Luciferase Reporter Assay 3′UTR sequences coding putative miRNA response element (MRE) for hsa-miR-143-3p in *KRAS*, *FGF1* and *FGF9* 3′UTRs, flanked by 30 nt on each side, were cloned into pmiRGLO vector. For rescue experiments, 4 point mutations were introduced to the MRE region during the oligonucleotide synthesis step to abolish the miRNA-mRNA interaction.

### 4.6. Proliferation Assays

After transduction and puromycin selection, 2 × 10^4^ cells per well were seeded on 96-well plate in 100 µL of culture medium. After 0, 24, 48, 72, and 96 h, 10 µL of Cell Counting Kit 8 (Sigma Aldrich, Bornem, Belgium) reagent was added to each well. Cells were incubated with CCK8 reagent for 4 h in 37 °C. Absorbance was measured on the GloMax-Multi+ Detection System (Promega, Madison, WI, USA). Each experiment was conducted in three technical and three biological replicates. The growth rate for each replicate in each time point was calculated as a fold change of absorbance in the first tested time point (0 h). Statistical significance was calculated with two-way ANOVA test or miR-143-3p expression as independent variable.

### 4.7. Dual Luciferase Reporter Assays

Predicted miRNA-mRNA interactions were validated with Dual-Glo Luciferase Reporter Assay (Promega, Madison, WI, USA). HEK 293T cells were seeded on 24-well culture plate 24 h before transfection. Cells were subjected to transfection at 60% to 80% confluency using JetPrime DNA/siRNA Transfection Kit (Polyplus Transfection, New York, NY, USA) to enable co-transfection with miR-143-3p coding or empty pCDH vector and pmiRGLO plasmids (Promega), containing 3′UTRs of the selected target genes. 250 ng and 50 ng of pCDH and pmiRGLO plasmid, respectively, was added per well. Luciferase activity was measured with GloMax-Multi+ Detection System (Promega) after 72 h from transfection. All experiments were performed in four replicates. A significant decrease in luciferase activity relative to control was indicative of direct interaction between the seed sequence of the miRNA (defined as the nucleotides at position 2–7 of the 5′ end of mature miRNA sequences) and the MRE in the 3′UTR of target mRNA.

## 5. Conclusions

miRNA-seq failed to reveal major differences between AYA and pediatric T-ALL patients. This is in line with the reports from other high-throughput analyses, indicating that the landscape of aberrations in AYA cases is at the interphase of those observed in pediatric and adult patients. The integration of multiomics data obtained in AYA patients seems to be another step to take. This approach might prove more powerful for the characterization of the molecular landscape of this leukemia in AYA patients and, more importantly, for the identification of AYA-specific, therapeutically targetable features. However, the heterogeneity of many cancers, including T-ALL, which is increasingly evident with the cumulation of omics and multiomics data, indicates another possible direction in the development of personalized therapy. The aim should be the identification of subgroups of patients, sharing the same molecular features (e.g., dysregulated signaling pathway), who might benefit from the same targeted therapy, regardless of the patients age, immunophenotypic or histological subtype and even regardless of the type of cancer diagnosed. The identification of patient subsets sharing the same driver aberrations and targetable lesions will enable drug repurposing and development of combination therapeutic strategies. In this respect, the identification of miRNAs contributing to the dysregulation of key signaling pathways is a line of basic research with potential future implications for the development of novel therapeutic options.

## Figures and Tables

**Table 1:** miRNAs differentially expressed in AYA T-ALL vs. pediatric T-ALL.

miRNA ID	Average Number of Normalized Reads	LogFC	*p* adj
hsa-miR-6724-5p	19	−4.411	0.013
hsa-miR-143-3p	6042	1.677	0.011
hsa-miR-151a-3p	1979	2.482	0.002
hsa-miR-4420-3p	1.64	4.461	0.005
hsa-miR-4728-3p	0.26	2.486	0.046
hsa-miR-4749-3p	0.67	3.787	0.005
novel_miRNA_chr17:7306866-7306887	0.49	2.696	0.029

logFC, fold change in log scale (for comparison of AYA vs. pediatric T-ALL); *p* adj, *p* value adjusted for multiple testing with Benjamini and Hochberg method with 0.05 significance level.

**Figure A1:**
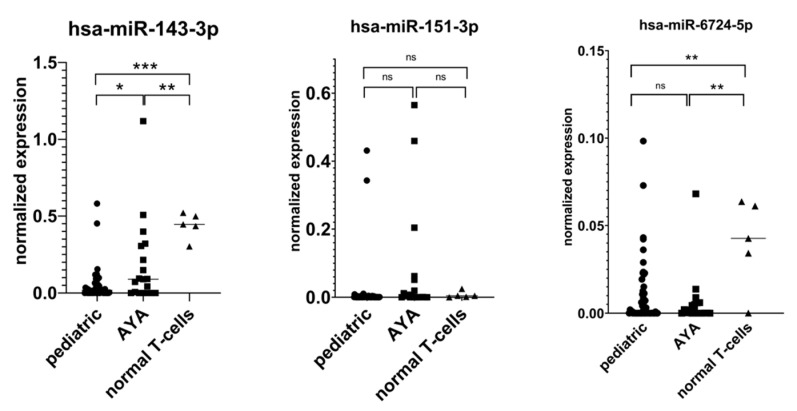
RT-qPCR validation of miRNA-seq results: * *p* < 0.05; ** *p* <0.01; *** *p* < 0.001; ns (not significant).

**Figure A2:**
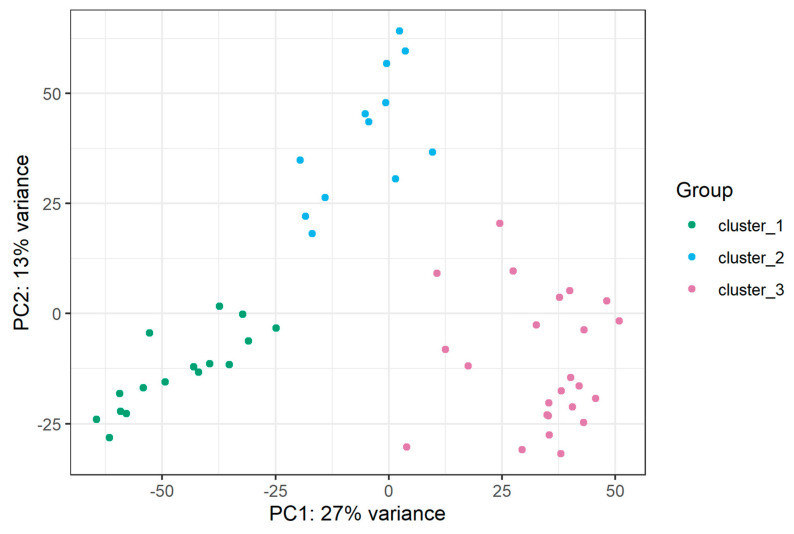
PCA plot based on mRNA sequencing presenting grouping of samples into clusters.

**Figure A3:**
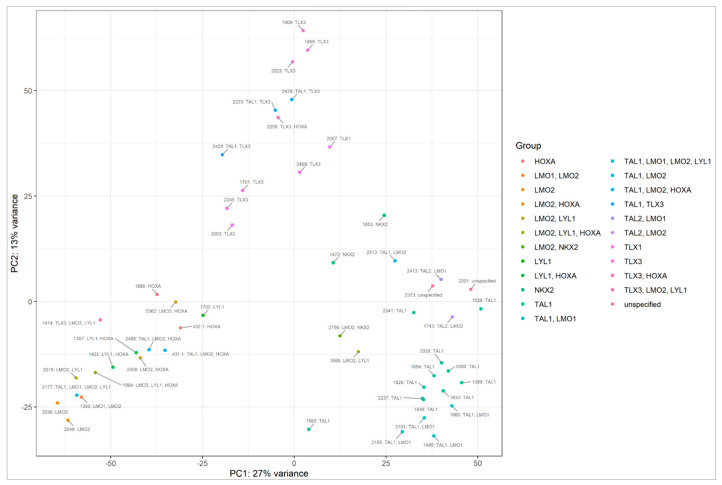
PCA plot based on mRNA sequencing presenting patients, classified into genetic subtypes, grouping into clusters.

## Data Availability

Small RNA-seq results of samples from batch I (T-ALL patients aged < 18 and controls) previously published [9] is available in the ArrayExpress database (http://www.ebi.ac.uk/arrayexpress) under accession number E-MTAB-7446. Small RNA-seq results of samples from batches II and III (pediatric and AYA patients) are available in the ArrayExpress database under accession number E-MTAB-11987.

## Supplementary Materials

The following supporting information can be downloaded at: https://www.mdpi.com/article/10.3390/ijms231710117/s1.

## Appendix A. RT-qPCR Validation of miRNA-Seq Results

For RT-qPCR validation, we selected three miRNAs based on the highest average read counts in miRNA-seq, namely: hsa-miR-143-3p, hsa-miR-151-3p, and hsa-miR-6724-5p. The expression levels of the remaining four miRNAs were low, precluding their detection by RT-qPCR. For hsa-miR-151-3p, RT-qPCR failed to validate our miRNA-seq results. For hsa-miR-6724-5p, we showed its downregulation in AYA vs. controls yet failed to confirm its lower expression in AYA vs. pediatric patients. In the case of miR-143-3p, we confirmed that it is highly expressed in AYA vs. pediatric samples, and we showed that its expression is downregulated in both age groups of T-ALL patients as compared to controls, indicating its potential tumor suppressor role in T-ALL.

## Appendix B. miRNAs Differentially Expressed in T-ALL Subtypes

Patients with available data on the immunophenotype of leukemic cells at diagnosis were classified into four immunophenotypic EGIL subtypes. miRNAs differentially expressed between EGIL subtypes are presented in Table S3. Patients with available data from mRNA-seq were classified into genetic subtypes based on aberrant expression/fusion of driver oncogenes. Patients characteristics is presented in Table S1. The PCA plot based on mRNA-sequencing showed grouping of patients into three clusters C1–C3.

Cluster C3 is mainly composed of samples classified into the *TAL1* subtype, and it includes all three samples showing *NKX2* expression. Cluster C2 is mainly composed of samples from the *TLX3* subtype, while cluster C1 is enriched in *LMO1*, *LMO2*, *LYL1* and *HOXA* cases. miRNAs differentially expressed between clusters C1, C2, C3, enriched in specific genetic subtypes are presented in Table S3.
