# Supplementary material for: Small RNA-Seq Reveals Similar miRNA Transcriptome in Children and Young Adults with T-ALL and Indicates miR-143-3p as Novel Candidate Tumor Suppressor in This Leukemia
Source: Int J Mol Sci. 2022 Sep 4;23(17):10117. doi: 10.3390/ijms231710117 (PMC9456032; doi:10.3390/ijms231710117)
Supplement: Supplementary file 1 [file ijms-23-10117-s001.zip › SUPPLEMENTARY MATERIALS.pdf]

SUPPLEMENTARY TABLES AND FIGURES

Small RNA-Seq Reveals Similar miRNA Transcriptome in Children and Young Adults with T-ALL and Indicates miR-143-3p as Novel Candidate Tumor Suppressor in This Leukemia

Małgorzata Dawidowska [1*], Natalia Maćkowska-Maślak [1], Monika Drobna-Śledzińska [1], Maria Kosmalska [1], Roman Jaksik [2], Donata Szymczak [3], Małgorzata Jarmuż-Szymczak [1,4], Alicja Sadowska-Klasa [5], Marzena Wojtaszewska [4#], Łukasz Sędek [6], Tomasz Wróbel [3], Jan Maciej Zaucha [5], Tomasz Szczepański [7], Krzysztof Lewandowski [4], Sebastian Giebel [8], Michał Witt [1]

SUPPLEMENTARY TABLES

Table S1

Characteristics of samples

Table S2

Statistics regarding miRNA-seq in the study cohort

Table S3

miRNA expression results in the study cohort

Table S4

Target prediction and overrepresentation analysis results

SUPPLEMENTARY FIGURES

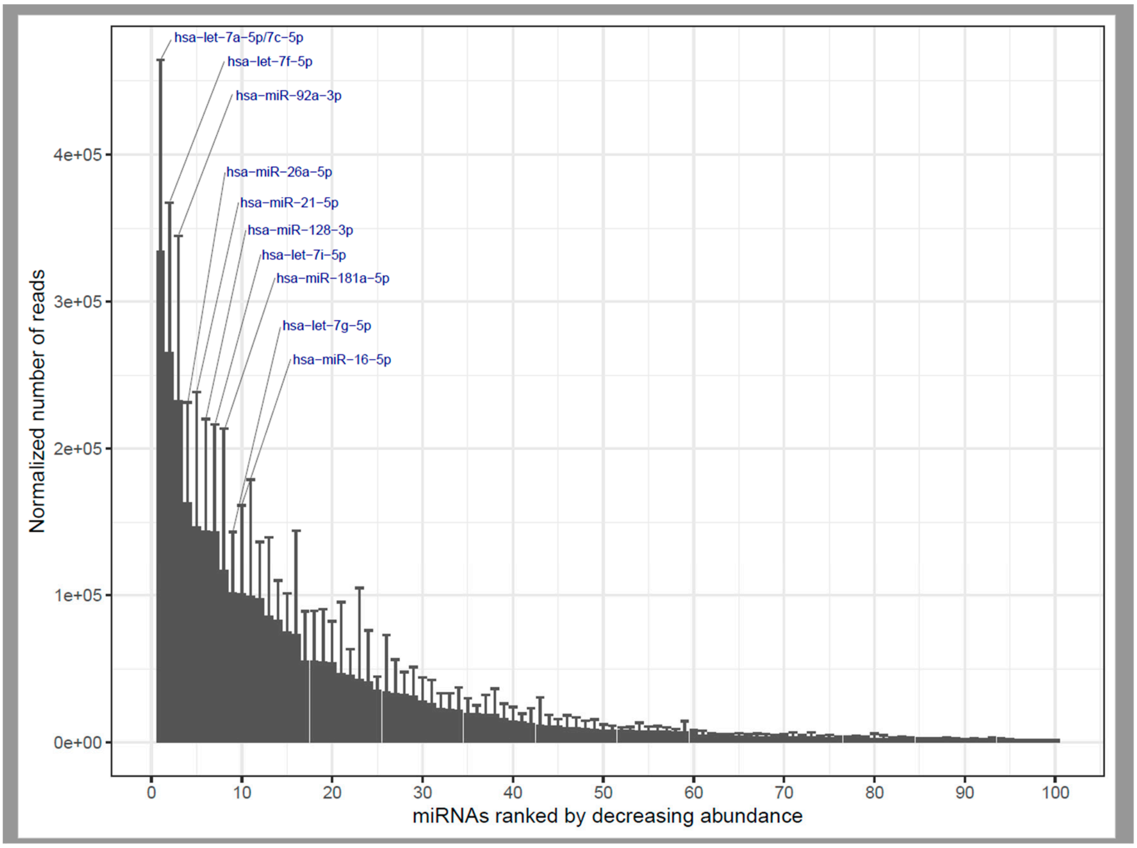

Figure S1. The 10 most highly expressed miRNAs in AYA patients

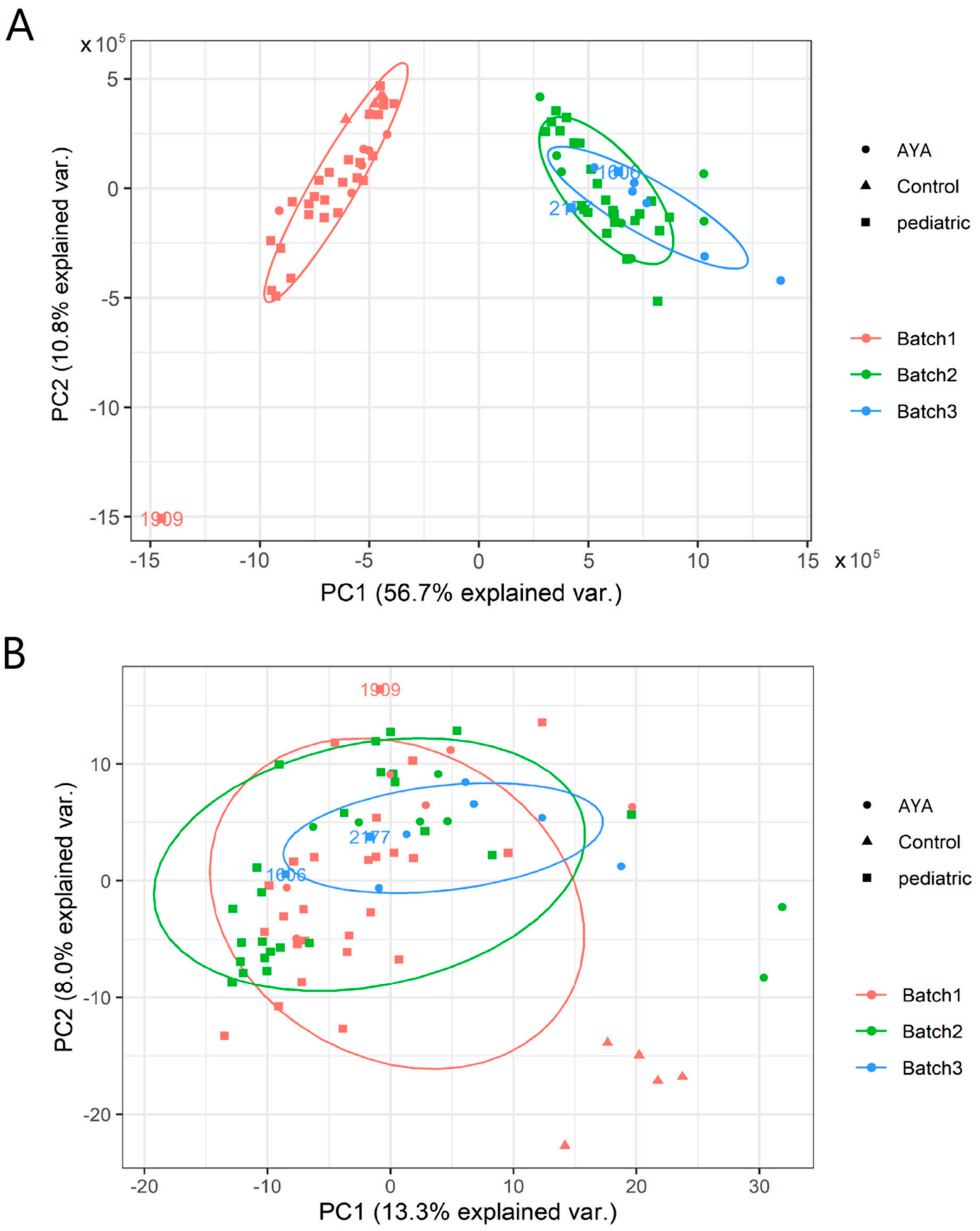

Figure S2. Principal component analysis plot based on miRNA-seq.

A/ PCA plot before the correction for batch effect. B/ PCA plot after the correction for batch effect based on ComBat.
